# Myosin Light Chains in the Progression of Cancer

**DOI:** 10.3390/cells13242081

**Published:** 2024-12-17

**Authors:** Savannah L. Kozole, Karen A. Beningo

**Affiliations:** Department of Biological Sciences, Wayne State University, Detroit, MI 48202, USA; kozolesa@wayne.edu

**Keywords:** Myosin light chain, metastasis, proliferation, cell mechanics, EMT, CAFs

## Abstract

The myosin light chains (MLCs) of non-muscle myosin II are known to regulate cellular architecture and generate cellular forces; they also have an increasingly emerging role in the progression of cancer. The phosphorylation state of the myosin light chains controls the activity of myosins that are implicated in invasion and proliferation. In cancers, when proliferation is greatly increased, cytokinesis relies on phosphorylated light chains to activate the contractile forces used to separate the cells. Likewise, during metastasis, kinase pathways culminate in aligning MLC structures for enhanced cell motility through stress fiber contraction and the accumulation of myosin filaments at the leading edge. This review summarizes the myosin light chain family members known to promote cancer progression and evidence of how their altered activities change the behavior of cells involving the mechanical-based processes of proliferation and cell movements during metastasis. In addition, myosin light chains impact the immune response to cancers and currently serve as biomarkers in staging this disease; a brief summary of these topics is provided at the end of the review.

## 1. Introduction

Cancer is a complex disease directed not only by biochemical signals but also by physical and mechanical information. Beginning at the initial stages of tumor development and progressing to extravasation and the colonization of distant organs, cancer cells communicate with their surroundings. Information obtained as mechanical and biochemical signals is translated into behavioral changes. Mechanical signals occur within the cell and, more interestingly, originate from the cell’s external microenvironment; such signals are often referred to as inside-out and outside-in signaling [1]. This crosstalk between cancer cells and mechanical properties in the tumor microenvironment (TME) remains poorly defined. A highly generalized view is that changes in the cytoskeletal architecture of cells lead to mechanical alterations in cell stiffness and changes in shape that activate key signaling pathways and downstream effectors that eventually mediate metastasis [2]. These biomechanical signals are transmitted bidirectionally through receptors, the extracellular matrix (ECM), and cytoskeletal components. It is these molecular interactions that impact cell-cycle progression and gene transcription, leading to the aberrant proliferation of cancer cells. Likewise, the multistep process of metastasis exploits mechanical forces for the mesenchymal transition and the generation of migratory forces and remodeling forces that occur in the tumor microenvironment. With the help of mechanical and biochemical factors, tumor cells leverage a niche tumor microenvironment built for proliferation and metastasis [3,4].

Clearly, contractile elements are necessary for the generation of biomechanical forces. In normal and disease states, NMII (non-muscle myosin II) is an essential component of the production of cellular force. NMII contains two heavy chains stabilized by two myosin light chains (MLCs), a coiled-coil tail region, and a non-coiled tail region responsible for the subcellular localization of NMII, with muscle myosin having a differential tail sequence that is optimized for muscle contraction [5,6,7]. NMII exists in its assembly-incompetent formation (10S) when unphosphorylated, with head-to-tail interactions, preventing the formation of bipolar filaments.

In non-muscle cells, MLCs are either regulatory (RLC) or essential ELCs. The movement of bipolar filaments is controlled by the phosphorylation of the regulatory light chain (RLC) on Ser^19^ and Thr^18^ residues, resulting in an increase in ATPase activity and increased NMII activity [8]. Controlling the functionality of RLCs is crucial for cell behavior and tumor formation [9]. The human RLC gene family encompasses *MYL2* (myosin regulatory light chain 2), *MYL5* (myosin light chain 5), *MYL9* (myosin regulatory light polypeptide 9), *MYL10* (myosin regulatory light chain 10), *MYL11* (myosin regulatory light chain 11), *MYL12A* (myosin regulatory light chain 12A), and *MYL12B* (myosin regulatory light chain 12B), with the ELC gene family including *MYL1* (myosin light chain 1/3, skeletal muscle isoform), *MYL3* (myosin light chain 3), *MYL4* (myosin light chain 4), and *MYL6* (myosin light polypeptide 6) [10].

The RLC gene family (*MYL9*, *MYL12A*, or *MYL12B*) is known to associate with unconventional myosin heavy chains (MHCs) including *MYH9* (myosin heavy chain 9), *MYH10* (myosin heavy chain 10), *MYH14* (myosin heavy chain 14), *MYH15* (myosin heavy chain 15), *MYH18* (myosin heavy chain 18), and *MYH19* (myosin heavy chain 19) [10]. The ELC gene family can associate with unconventional myosins such as *MYH1* (myosin heavy chain 1), *MYH5* (myosin heavy chain 5), *MYH6* (myosin heavy chain 6) which is specific to cardiac myosin, and *MYH7B* (myosin heavy chain 7B) which is a member of the sarcomeric family [11]. Two important caveats to these associations are: (1) RLCs do not exhibit specificity for MHCs, as they are capable of interacting with various myosins or functioning independently, and (2) RLCs display promiscuity in terms of their tissue and cell-type specificity, meaning their expression and function can vary across different biological contexts [12]. For example, an interaction analysis revealed that *Myl9*, *Myl12a*, and *Myl12b* are associated with MHCs such as *MYH6* and *MYH7* in cardiac myosins, *MYH1*, and *MYH2* in skeletal muscle, as well as the non-muscle myosins *MYH10* and *MYH9*, as denoted above [13]. The integrity of myosin II relies upon RLCs, which are responsible for the stability of conventional non-muscle MHCs. In one instance, the depletion of *Myl12a* and *Myl12b* in NIH3T3 fibroblasts, resulted in a decrease in *Myh10* and *Myh9* levels [13]. Thus, RLC expression is required to maintain intact MHCs.

This review will not delve into the intricacies of MHCs and cancer, as this would be redundant [14]. Many previous studies have focused on different members of the MHC superfamily while neglecting the influence of MLCs, this review will focus on RLCs in the cellular acquisition of malignant properties [15]. We will summarize recent information on mutations and altered expression of MLC genes in driving the progression of cancer in the cell cycle and in the diverse stages of metastasis. Finally, we discuss the use of MLCs as predictive biomarkers in the staging of the disease and their involvement in stimulating the immune response.

## 2. Cell Cycle: The Contribution of MLCs to Cancer Proliferation

**Figure 1:**
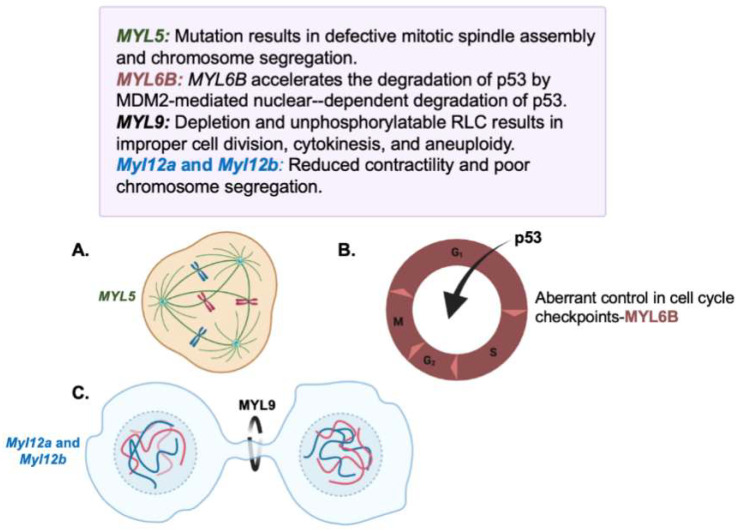
Myosin Light Chain and the Tumor Cell Cycle. Myosin light chains act in the cell cycle, controlling proliferation. (**A**) *MYL5* localizes to the spindle poles in early mitosis (G1/S) and remains until mitotic exit (G2/M) through its association with Myosin X *(MYO10)* [16]. (**B**) *MYL6B* is a tumor promoter which degrades p53 to cause aberrant control of cell cycle transitions leading to hyper proliferation and DNA damage [20]. (**C**) The contractile ring is regulated by Myl9 and Myl12a/Myl12b; however, Myl9 expression also extends or shortens G0/G1, controlling tumor re-initiation as well as proper cell division. This force is generated by NMII17. Figure made in Biorender.

The acquisition of a cancerous phenotype includes general hallmarks relevant to myosin activity. Cancer cells undergo clear morphological changes and an uncontrolled rate of cell division. Previous studies have implicated RLCs in tumorigenesis and the promotion of tumor formation due to poor spindle formation, cytokinesis failure, and DNA instability (Figure 1). For example, when depleted, the RLC *Myl5* led to improper assembly of the mitotic spindle and defective chromosome segregation and congression [16]. As *Myl5* expression is correlative to prolonged mitotic activity, *Myl5* can contribute to tumor formation purely from prolonged mitotic checkpoint activation. Indeed, the Myl5 expression levels remain constant throughout the cell cycle, changing its subcellular localization dependent upon cell-cycle progression.

One of the most important contractile activities in cells occurs during cytokinesis. Following chromosome segregation, cytokinesis divides the cell into mother and daughter. Failure to properly execute cytokinesis can result in the production of tetraploid cells. Phosphorylation of RLC is required for cytokinesis, and overexpression of non-phosphorylated RLC is known to lead to cytokinesis failure. Indeed, various cancer cell lines exhibit lower levels of RLC phosphorylation, linking lower levels to cancer and failed cytokinesis [17].

The actin cortex assists in cell division by providing mechanical forces driven by NMII. Its regulation is controlled by an upstream cascade of phosphorylation events involving RLCs, ROCK, and MLCK, which, in turn, control NMII recruitment and NMII turnover, respectively. As upstream targets of these kinases, *Myl12a* and *Myl12b* knockdown results in a significant reduction in cellular contractility and poor chromosome segregation [18]. The contractile function delivered by myosin II aids in the release of adhesions, the rounding of cells, which is critical to cleavage furrow formation, and the assembly of the contractile ring. Furthermore, the RLC *Myl9* and depletion of its phosphorylated form result in a loss of actomyosin contractility in the cell cortex during mitosis. An upstream regulator of *Myl9* that is formally known as Junb also leads to abnormal cellular motility and contractility when depleted [19]. Only the expression of *Myl9* resulted in the rescue of migratory ability and stress fiber formation in several highly contractile cell types [19]. Failure of these processes results in aneuploidy, improper cell division, and cytokinesis failure [17,19].

Myosin light chain genes have been shown thus far to have positive effects on maintaining cellular structure and proper cell division; one exception is *MYL6B*, which has deleterious effects on normal cell function. *MYL6B* acts upon the tumor suppressor gene p53, which functions in normal cells to prevent the division of damaged cells by arresting the cell cycle and promoting the subsequent apoptosis of irreparable cells. Mechanistically, *MYL6B* regulates the MDM2-P53 pathway, which is essential for cell adhesion, migration, and endocytosis. *MYL6B* was found to facilitate the binding of MDM2 to p53, with overexpression of *MYL6B* resulting in increased co-precipitation of p53 protein with MDM2 in a dose-dependent manner. This increased binding affinity, leading to *MYL6B*-mediated ubiquitination and the degradation of p53 by regulating endogenous levels of the protein [20]. It is understood that MDM2-mediated p53 ubiquitination promotes p53 degradation through the proteosome, but without co-distribution of *MYL6B* with the p53 protein or MDM2 in the cytoplasm [20]. This led to the discovery that *MYL6B* accelerates the degradation of p53 through the MDM2-mediated nucleus-dependent degradation of p53. In addition, *MYL6B* can work with myosin holoenzymes (MYH9 and MYH10) to accelerate the degradation of p53 [20]. In addition to controlling tumor suppressor genes, RLC *Myl9* can be targeted to understand how cells re-initiate tumors after exiting a long G1 phase [18,21]. *Myl9* overexpression or deletion causes an extended or shortened G0/G1 phase. As cells are usually resistant to chemotherapy at this stage, *Myl9* can cause the exit of dormant cells into the G1 phase [18,21].

## 3. MLCs in Invasion and Metastasis

**Figure 2:**
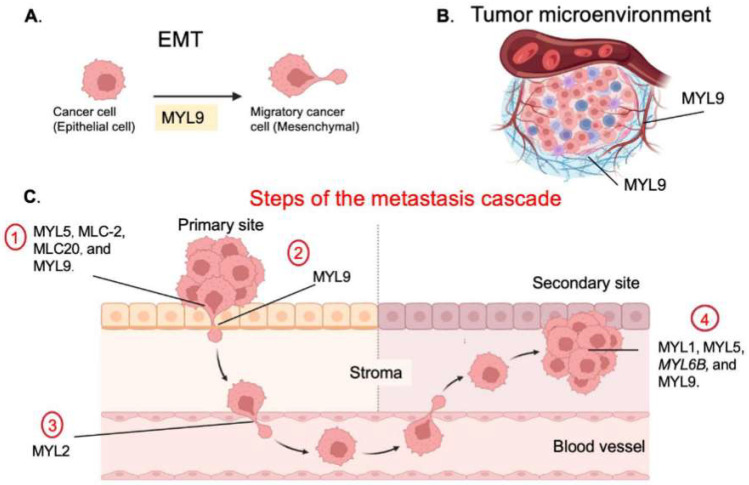
Myosin light Chain and Metastasis. Myosin light chains have been implicated in various steps of the metastasis cascade. (**A**) Most often, depending upon the tumor origin type, tumors will acquire a more invasive phenotype by upregulation of invasive characteristics to enter the stroma and surrounding circulatory system. MYL9 expression is linked to the EMT process and responsible for the signaling pathways involved in such phenotypic changes [22,23] (**B**) A tumor microenvironment (TME) is a sophisticated niche supplying the tumor with vasculature, a process known as angiogenesis. MYL9 is critical for angiogenesis by activating actomyosin contractility and angiogenic sprouting. Highly contractile cells, such as cancer-associated fibroblasts, in the TME can remodel the ECM and promote metastasis. *MYL9* supports the CAF phenotype [24,25,26]. (**C**) 1. Invasive cancer cells acquire various mechanisms for translocation and penetration through the stromal matrix and myosin light chains MYL5, MLC-2, MLC20, and MYL9 help cancer cells gain invasive properties through mechanotransduction and control of genetic factors [21,24,25,27,28,29,30]. 2. For metastasis to occur, a cancer cell must break through the tumor basement membrane, MYL9 assists in this activity by enhancing actomyosin contractility and MMP degradation [24,31]. 3. After cancer cells deadhere from the tumor, MYL2 helps to increase dissemination and intravasation into surrounding vasculature [26]. 4. A cancer cell must then adhere to blood vessels and extravasate or embolize the vessel to begin colonizing the secondary site. Finally, MYL1, MYL5, MYL6B, and MYL9 promote metastasis in several cancers discovered by in vivo assays [18,20,32,33,34,35,36]. Figure made in Biorender.

Metastasis is a multi-stage process that requires several complex and sequential changes to occur within cancer cells. These changes happen at each stage, or cancer cells may arrest or die before successful colonization of a secondary tumor site occurs. Due to the actomyosin-generated forces that contribute to tumor progression, the MLC pathway is activated during cancer cell invasion and other stages of metastasis (Figure 2). A cancer cell has the capacity to break through the tumor basement membrane and establish a mesenchymal phenotype to metastasize. Once cancer cells enter the stroma, they collaborate with stromal cells to remodel the ECM that lies in their path as they are drawn to the vasculature. Once the circulation is intravasated, the process essentially moves in reverse. Below, we have highlighted studies in which MLCs have been implicated in specific stages of metastasis, with a particular emphasis on the invasive movements regulated by MLCs.

### 3.1. Breaching the Basement Membrane and the EMT

Active myosin II contractility is required for force-dependent breaking of the basement membrane barrier [37]. This mechanism is initiated by the acquisition of invasive structures and myosin II contractility, translocating the cell body through the basement membrane, adhering to integrins, and pulling the collagen inward [37]. This phenomenon was also discovered in a breast cancer cell line where actomyosin contractility was used to expand the cell body, pushing the cell through the basement membrane [22]. However, before cancer cells breach the membrane, they will break off from the primary tumor to invade the surrounding stroma. To break free, cancer cells may break cell–cell contacts and acquire a more invasive phenotype, and *MYL9* is thought to control the transition from the epithelial to the mesenchymal phenotype. To do so, *MYL9* can increase N-cadherin while decreasing E-cadherin. One potential mechanism involves the transcription factors associated with the EMT process, such as ZEB1, which can directly bind to the *MYL9* promoter in cancer-associated fibroblasts (CAFs). This binding enhances the expression of *MYL9*, thereby influencing tumor biology and EMT dynamics. Notably, silencing *MYL9* results in reduced N-cadherin and vimentin expression, accompanied by an upregulation of E-cadherin levels, suggesting a shift in cellular phenotype and modulation of EMT-related processes [23]. In gastric cancer, *MYL9* has been associated with the EMT process based on its increased expression vs. other prognostic factors, such as MSI, MSS/TP53+, and MSS/TP53 [38]. Specific signaling pathways correlated with the EMT, further, apical junction were also found to be compromised by activated pathways of *MYL9* [38]. For cancer cells to exist in a solid tumor mass, *MYL9* will increase the epithelial characteristics, such as increased E-cadherin and decreased N-cadherin levels, decreased invasive capabilities, and increased cell–cell contacts. MMP-9, part of the matrix metalloproteinase family, can cleave and degrade basement membrane type IV collagen to induce the invasion and metastasis of cancer cells. High expression of *MYL9* is associated with increased levels of MMP-9, and knockdown of the gene causes a decrease in the expression of MMP-9 protein [24].

### 3.2. CAF Stromal Cells

CAFs that are embedded in the tumor stroma work to remodel the ECM and encourage metastasis. Two mutually independent pathways, MRTF-SRF and YAP-TEAD, are transcriptionally activated in response to mechanical forces and contractile activity. The MRTF-SRF pathway is upregulated by cytoskeletal regulators such as *MYL9*. The targets of MRTF are more prevalent with the increase in cancer progression from normal fibroblast cells to CAFs, and *MYL9* expression is increased in CAFs in colorectal cancers [25]. CAFs possess signaling receptors that aid in maintaining the CAF phenotype, which is important for tumor progression, angiogenesis, and shaping an immunosuppressive environment. *MYL9* can regulate the secretion of TGF-β1, thereby modulating the immune microenvironment across various tumors. Additionally, *MYL9* influences protein expression in CAFs, promoting enhanced ECM remodeling and stiffness. This results in a positive feedback loop that sustains CAF activation, which, in turn, further impacts tumor biology and the progression of EMT processes [23]. Further data revealed that *MYL9* overexpression in normal fibroblasts (NFs) increased matrix remodeling and invasion, promoting the importance of the function and maintenance of the CAF phenotype [26,39].

CAFs can also assist tumor cells’ entrance and exit from the vasculature. A pro-metastatic factor known as 12(S)-HETE can control stromal cells, such as CAFs, to enable cooperation and facilitate the dissemination of metastases and intravasation into lymphatic vessels. The pro-metastatic factor 12(S)-HETE can act as an endothelial retraction factor and enhance vascular permeability, making it indispensable for cancer metastasis. The 12(S)-HETE signal can activate Myl2 for endothelial junction retraction, CAF contractility and mobility, and circular chemorepellent-induced defects (CCIDs), which are prerequisites for tumor cells’ entry into the vasculature [40].

### 3.3. Invasion

Cancer cells can adapt modes of invasion broadly as single cells or through collective migration. Single-cell invasion can be further categorized into ameboid invasiveness and mesenchymal invasiveness, which are characterized by stress fibers, elongated cell morphology, adherence to the ECM, a polarized leading edge with actin-rich protrusions, and ECM degradation [41]. In migrating cells, there are several types of dynamic protrusions, including filopodia, lamellipodia, and invadopodia. These protrusions are responsible for sensing the environment and stabilization through integrin-mediated cell attachment to the ECM. MLC genes are known to increase the invasive abilities of cancer cells, and they are found at the leading edge of cell protrusions [24,27,32,39].

Two general examples of protrusion disruption involve *MYL11* and *MYL5*. It was reported that the knockdown of *Myl12a* and *Myl12b* in fibroblasts changed the morphology and impaired the formation of actin fibers [13]. Knockdown of these myosin isoforms resulted in increased cell size and protrusions of filopodia from the cell body and cytoplasm, thus causing a shift in actin dynamics. Indeed, the RLC *Myl5* is also important for actin polymerization dynamics, as seen with *MYL5* colocalization to the leading cell edge of filopodia. Myl5 mutants can promote metastasis in vivo, which is determined by the overexpression of *MYL5* in cervical cancer cells. This overexpression resulted in popliteal lymph node and liver metastatic nodules in comparison with *MYL5*-silenced groups and a remarkable decrease in metastatic lesions by over 50% [27]. Disruption of either of these MLCs impacts cell invasion in a negative manner.

**Figure 3:**
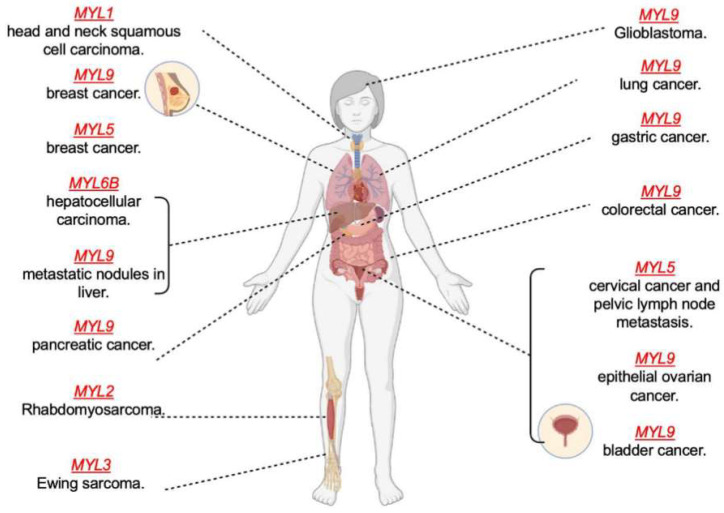
Myosin Light chain in various cancer(s). Myosin light chains are implicated in causing cancer in different organs and tissues. From left to right, *MYL1* promotes tumor metastasis in head and neck squamous cell carcinoma (HNSCC) and is an unfavorable prognostic marker [33]. Reduction in *MYL9* is linked to better overall survival (OS), post progression survival (PPS), and relapse free survival (RFS) in breast cancer patients [34]. Low expression of *MYL5* worsens the pathological stage and prognosis in breast cancer patients [34]. *MYL6B* is a tumor driver gene in hepatocellar carcinoma (HCC) and promotes the degradation of p53 tumor suppressor by MDM2 mediated ubiquitination [20]. *MYL9* promotes liver metastases in vivo in late-stage colorectal cancer (CRC) [14]. The pattern of *MYL9* expression in pancreatic cancer (PDAC) tissues correlates with poor prognosis in OS and distant metastases free survival [18]. *MYL2* is involved in the pathogenesis of rhabdomyosarcoma and can aid in reducing mortality rates [42]. *MYL3* expression levels are correlated with poor OS in patients with Ewing sarcoma [43]. High MYL9 expression is correlated with poor prognosis in newly diagnosed Glioblastoma patients and this expression is indicative of tumor aggressiveness [32]. *MYL9* correlates with faster progression in non-small lung cancer patients [34]. Reduced MYL9 is significantly linked to better OS, PPS, and RFS in gastric cancer patients [34]. *MYL9* upregulation is linked to early onset colorectal cancer. MYL5 is linked to cervical cancer and pelvic lymph node metastases [27]. *MYL9* high expression is linked to poor prognosis of epithelial ovarian cancer and bladder cancer [44]. *MYL9* is linked to prostate cancer, as decreased expression of *MYL9* leads to malignant progression and the downregulation of *MYL9* is linked to better OS and biochemical free recurrence-free survival in prostate cancer [45]. Figure made in Biorender.

MLC genes such as *MLC-2* are overexpressed in several cancers, including melanoma and pancreatic–ductal adenocarcinoma (PDAC) (Figure 3). *MLC-2* overexpression reduced cellular traction force, while its downregulation increased cytoskeletal stiffness, traction force, and invasion of PDAC cells through retinoic acid receptor B, a nuclear receptor that, when dysregulated, accompanies the early stages of cancer [21]. In some cases, overexpression of MLC genes has led to a significant increase in the invasive abilities of cancer cells, as revealed by Matrigel invasion assays of the RLC *MYL5* in comparison with control conditions [27]. For example, cervical cancer (CC) patients exhibit bidirectional regulation from the HIF-1 transcription factor [27]. *MYL5* can induce gene expression in cervical cancer by binding to the AGCTCC promoter by the HIF-1 start site, upregulating HIF-1 in CC, and increasing cells’ invasive abilities [27].

There is much focus on *MYL9* due to its influence on cancer-causing pathways and its ability to be a prime candidate for therapeutics to treat metastatic cancer by targeting its invasive ability. *MYL9* can act as either a metastatic suppressor or inducer depending on its expression level. The human atlas genome demonstrates that *MYL9* is lowly expressed in several metastatic cancer cell lines, suggesting a role as a metastatic suppressor. For example, *MYL9* was found to be downregulated in response to mechanical stimulation in an in vitro mechanical invasion assay [46]. Thus, to enhance the mechanical response and increase invasion, *MYL9* must be downregulated. Therefore, if *MYL9* is overexpressed, this would inhibit the invasive response to mechanical cues. *MYL9* expression is high in several non-metastatic cancer cell lines and displays differential expression depending on the tumor type and the progression of cancer. This differential expression leads to different survival outcomes in various cancers. *MYL9* expression promotes invasion in breast cancer cell lines and a hypermotile phenotype, and it is localized to the invasion edge of tumor cells, suggesting that *MYL9* is indeed involved in invasion-promoting factors in several cancers [24,28,29,32].

The *MYL9* axis can regulate both protein activity and cellular function by modifying signaling pathways and post-translational modifications (PTMs) that have deleterious downstream effects on certain cancer types. *MYL9* plays a key role in early-onset colorectal cancer (CRC) by influencing the cGMP-PKG and oxytocin signaling pathways. It may help prevent the downregulation of immunosuppressive proteins involved in the cancer’s progression [47]. High expression of *MYL9* is known to be a tumor and metastasis promoter for several cancer subtypes, as found by quantifying metastatic nodules on livers and lymphatic metastases in CRC and non-small-cell lung cancer [15,23]. *MYL9* can promote metastasis by modifying the ubiquitin–proteasome system (UPS) through the stabilization and activation of E3 ubiquitin ligase PRPF19 and the regulation of serum-response factor (SRF) through myocardin-related transcription factor (MRTF). *MYL9* can activate other biochemical signaling pathways, such as HIPPO and NOTCH, to induce tumorigenesis. *MYL9* can combine with YAP1, a vital regulator of the HIPPO pathway that is crucial for promoting cancer development, especially in CRC, to activate HIPPO signaling and promote the proliferation of cancer cells [24].

### 3.4. MLCs, Mechanical Communication, and the Stroma

The tumor microenvironment is a complex niche exposing metastatic cells to numerous mechanical stimuli that drive their behavior. Cells perceive these diverse stimuli at the cell membrane and translate them into biochemical signals that can activate tumorigenic pathways by utilizing physiological forces or by destabilizing important homeostatic mechanisms. Cell–ECM adhesions transduce the force from cells to the external environment and vice versa. As central molecules in mechanotransduction pathways, myosins and their inactivation by the general inhibitor blebbistatin result in the loss of F-actin stress fibers, abnormal focal adhesions, and elimination of cellular traction force in ovarian cancer cells [48]; however, treatment with blebbistatin did not perturb phospho-MLC accumulation with F-actin structures. Phosphorylation of MLC is important for the formation of focal adhesions (FAs) and stress fiber formation. When MLCK is inhibited in NIH3T3 cells, the numbers of early focal complexes are minimized [49]. Since blebbistatin did not affect the colocalization of phospho-MLC and F-actin, this suggests that upstream kinases of RLCs play a more significant role. Upstream of RLCs, the activation of RhoA and Cdc42 leads to sustained focal adhesions with increased P-MLC localization at the cell periphery and to stress fibers. Thus, this suggests that MLC levels maintain cellular integrity and focal adhesion complexes [49]. RLC depletion can interfere with focal adhesion formation and maturation, with endogenous RLC-depleted cells lacking elongated adhesions in the lamella, cell body, and nascent adhesions in the lamellipodium [8].

The ability of cells to sense their environment and respond by stiffening the matrix highlights a cancer cell’s ability to mechanically adjust its internal tension and the external ECM to increase its metastatic potential [48]. MLCs, coupled with their upstream regulator ROCK, influence cell–matrix tension. Stiffer substrates increased F-actin organization, focal adhesion formation, and MLC phosphorylation, which positively correlated with increased traction force, cell spreading, and an increase in metastatic potential. For example, myosin light chain phosphorylation in human epithelial ovarian cancer cells (EOCs) is regulated by ECM stiffness, as active, phosphorylated MLCs co-localized with F actin in cells on a stiff substrate (25 kPa) in comparison with a soft substrate (3 kPa) [48]. High ECM stiffness (10–30 kPa), depending on tissue type and disease progression, activates signaling pathways linked to cell migration and invasion [50]. This same phenomenon is true in mesenchymal derived tumors, as seen with softer tumors exhibiting reduced pMLC2 levels and mechanosignaling [51]. YAP1, a direct binding partner of *MYL9* linked to a pro-cancer signaling pathway, is transcriptionally activated when a cell senses a stiff ECM. This enhanced stiffness results in the activation of proto-oncogene Src (c-Src), a receptor tyrosine kinase linked to human cancers, and the subsequent activation of YAP and cancer progression, showing a correlative trend with myosin activity and mechanotransduction in metastasis [15].

## 4. MLCs in Immunity

The body’s anti-tumor response is an important process that allows the immune system to recognize and reject malignant cells. Immunotherapy and immunology are important approaches to cancer treatment. Approaches exploit the body’s innate armory of CD4+T cells, macrophages, and dendritic cells in addition to the antigen-specific immune response. MLCs have also impacted this aspect of cancer biology. *MYL1*, *MYL2*, and *MYL3* were shown to have a positive correlation between expression levels and the infiltration of CD4+T cells in head and neck squamous cell carcinoma (HNSCC). Macrophages, which are important for eliminating diseased cells, and dendritic cells (DCs), which can stimulate naive T cells into adaptive immunity, also showed a positive relationship between *MYL3* expression and infiltration levels [33]. *MYL1* has a strong positive correlation with central memory CD8 cells, central memory CD4+ cells, natural killer cells, mast cells, natural killer T cells, T-follicular helper cells, and regulatory T cells [33]. These cells play a role in adaptive immunity and the maintenance of immune homeostasis.

*MYL9* is an important driver of immunity, as myl12a/myl12b and myl9 are ligands for CD69, an important regulator of immune responses. myl9 is seen to form net-like structures that are formally known as myl9 nets, which allow CD69-expressing antigen-specific T cells to migrate to inflamed tissues, where they secrete cytokines and chemokines that drive and amplify the inflammatory response. As chronic inflammation or even low-grade systemic inflammation creates a perfect niche for the support of metastatic spread, these myl9 nets should be targeted for cancer therapies [52]. Most importantly, the interplay between the TME, immune factors, and effector molecules plays an essential role in the efficacy of cancer immunotherapy.

CAFs are known to directly regulate immune infiltration and the tumor immune microenvironment. It was found that *MYL9* can directly regulate cytokines and chemokine production in CAFs, affecting the immune microenvironment in CRC [23]. Expression of the *MYL9* gene is positively correlated with the expression of CAFs in BRCA, BLCA, cervical squamous cell carcinoma and endocervical adenocarcinoma (CESC), CHOL, COAD, STAD, esophageal carcinoma (ESCA), kidney renal papillary cell carcinoma (KIRP), and LIHC. *MYL9* was significantly correlated with the infiltration of CD8+T cells and CD4+T cells in COAD, neutrophils and DCs in lung adenocarcinoma (LUAD), and macrophages in BLCA, COAD, ESCA, KIRP, and LIHC [18]. It is important to note that *MYL9* was correlated with more immune markers in metastatic cancers than in normal tissues; ~60–70/80 were immune marker hits compared with ~20/70 in normal tissues [18]. These tumor-infiltrating lymphocytes (TILs) can initiate an anti-tumor response in several cancers and can serve as a biomarker for the efficacy of treatment.

## 5. MLCs as Biomarkers

Biomarkers serve to reduce premature mortality and provide the best possible outcome of therapies for cancer patients. The prognostic and immunological roles of several myosins have been studied due to the correlation between myosin light chains and cancer progression. The functional role of each myosin light chain differs depending on expression levels and the type of cancer. Differential expression of myosin light chain genes corresponding to poor survival, low survival rate, and high malignancy can aid in targeted drugs and therapeutics to reduce mortality. *MYL9* expression is lowered in cancer types such as breast cancer (BRCA), bladder cancer (BLCA), colon adenocarcinoma (COAD), esophageal squamous cell carcinoma (ESCC), CC, and stomach adenocarcinoma (STAD), and it is higher in cancers such head and neck squamous cell carcinoma (HNSCC), liver hepatocellular carcinoma (LIHC), cholangiocarcinoma (CHOL), and (GBM) glioblastoma, as documented in a cancer microarray database [29,32,34]. Kaplan–Meier plots revealed that cancer with lower or decreased expression of *MYL9* showed better overall survival (OS) and better post-progression survival in gastric, ovarian, and breast cancer [34]. However, whole-human-genome expression arrays revealed increased *MYL9* expression in pancreatic tissue in comparison with normal expression, indicating better disease- and metastasis-free survival and OS rates and demonstrating MYL9 as a novel biomarker for pancreatic ductal adenocarcinoma (PDAC) [18]. Low expression of other myosin light chains, such as *MYL2*, is correlated with poor prognosis in rhabdomyosarcoma patients; subsequently, the differentiation and proliferation in muscles were found to be abnormal, leading to the occurrence of tumors [42]. In a recent article, *MYL5* was demonstrated as a novel biomarker for breast cancer. The Omnicome database displayed that *MYL5* expression was significantly decreased in BRCA, head and neck squamous cell carcinoma (HNSC), lung adenocarcinoma (LUAD), and thyroid carcinoma (TC). There was also a markedly large difference in *MYL5* expression levels between pan-cancer and normal tissues, where distant recurrence-free survival, post-progression survival, relapse-free survival, distant metastasis-free survival, and OS of patients with breast cancer were longer and had better odds with high expression of *MYL5* compared with lower expression [35]. Breast cancer can broadly be divided into four main sub-groups: luminal A, luminal B, basal-like triple negative, and HER-2-enriched, ranging from the most aggressive to the most frequent rate of metastasis. In many cases, *MYL5* expression can prolong the overall survival and relapse-free survival in breast cancer patients with negative lymph node metastasis [35]. Since tumor reoccurrence is inevitable in some cancer types, survival after treatment is important to consider. For instance, high *MYL9* expression in tumor cells had poorer overall survival (OS) and recurrence-free survival in ESCC, demonstrating that *MYL9* is an independent factor affecting OS after curative treatment therapy [32]. In addition, increased expression of *MYL6B* lessens the survival time of patients after therapy [20].

## Data Availability

All information used for this review are cited in the references.
